# Supplementary material for: Reproducible disease phenotyping at scale: Example of coronary artery disease in UK Biobank
Source: PLoS One. 2022 Apr 5;17(4):e0264828. doi: 10.1371/journal.pone.0264828 (PMC8982857; doi:10.1371/journal.pone.0264828)
Supplement: S1 Table — (DOCX) [file pone.0264828.s001.docx]

**Supplementary Table 1:** **List of ICD-10 and ICD-9 terms used to define the MI phenotype**

| **Algorithm Step** | **ICD 9*** | **ICD 10** | **Other** | **Definition** |
| --- | --- | --- | --- | --- |
| I21 in EHR | 410.XX | I21.* |  | ST elevation (STEMI) and non-ST elevation (NSTEMI) myocardial infarction.  Further MI classification can be achieved by stratifying by ICD terms:  STEMI: I21.0, I21.1, I21.2, I21.3, I22.0, I22.1, I22.8  NSTEMI: I21.4, I21.9, I22.9 |
| Other MI codes in secondary care |  | I22.* |  | Subsequent ST elevation (STEMI) and non-ST elevation (NSTEMI) myocardial infarction |
|  | 429.79 | I23.* |  | Certain current complications following ST elevation (STEMI) and non-ST elevation (NSTEMI) myocardial infarction (within the 28-day period) |
|  | 411.0 | I24.1 |  | Dressler's syndrome (Postmyocardial infarction syndrome) |
|  | 412 | I25.2 |  | Old myocardial infarction |
| MI in death records |  |  |  | We retain events where any cause of death is listed as “I21” (MI) or “I252” (old MI). Events are then stratified as: “death1_MI” where I21 is the primary cause of death, or “death2_MI” where I21 is listed as a secondary cause of death. Where I252 appears in the mortality record, death1_oldMI is given when the code is in the primary position, and death2_oldMI where I252 is in any secondary position. It is possible for participants to have an I21 and I252 in any position of the death record. In this case, these are stored as separate events, although they will have the same date. |

**ICD 9 codes were not used in UKB but are made available for studies where they are still used*
