# Supplementary material for: Reproducible disease phenotyping at scale: Example of coronary artery disease in UK Biobank
Source: PLoS One. 2022 Apr 5;17(4):e0264828. doi: 10.1371/journal.pone.0264828 (PMC8982857; doi:10.1371/journal.pone.0264828)
Supplement: S2 Table — (DOCX) [file pone.0264828.s002.docx]

**Supplementary Table 2: List of EHR codes used to define the CAD phenotype**

| **Algorithm Step** | **ICD 10** | **ICD 9*** | **Other** | **Definition** |
| --- | --- | --- | --- | --- |
| UA in EHR | I20.0 | 411.1 |  | Unstable angina |
| Revasc in EHR | Z95.5 | V45.82 |  | Presence of coronary angioplasty implant and graft |
|  |  |  | K40 - 46 | Coronary Artery Bypass Graft (OPCS codes) |
|  |  |  | K75; K50; K49; K47.1 | Percutaneous coronary intervention (OPCS codes) |
| Other CAD in EHR | I20.1 | 413.1 |  | Prinzmetal angina |
|  | I20.8 | 413.0 |  | Angina decubitus |
|  | I20.9 | 413.9 |  | Angina pectoris NEC/NOS |
|  | I25.0 | 414.0X |  | Coronary atherosclerosis |
|  | I25.1 | 414.01 |  | Coronary atherosclerosis of native coronary artery |
|  |  | 429.2 |  | Cardiovascular disease, unspecified |
|  | I25.3 | 414.10 |  | Aneurysm of heart (wall), |
|  |  | 414.19 |  | Other aneurysm of heart |
|  | I25.4 | 414.11 |  | Aneurysm of coronary vessels |
|  |  | 414.12 |  | Dissection of coronary artery |
|  | I25.6 | 414.8 |  | Other specified forms of chronic ischemic heart disease |
|  | I25.7 | 414.02 |  | Coronary atherosclerosis of autologous biological bypass graft |
|  |  | 414.03 |  | Coronary atherosclerosis of nonautologous biological bypass graft |
|  |  | 414.04 |  | Coronary atherosclerosis of artery bypass graft |
|  |  | 414.05 |  | Coronary atherosclerosis of unspecified type of bypass graft |
|  |  | 414.06 |  | Coronary atherosclerosis of native coronary artery of transplanted heart |
|  |  | 414.07 |  | Coronary atherosclerosis of bypass graft (artery) (vein) of transplanted heart |
|  | I25.8 | 414.2 |  | Chronic total occlusion of coronary artery |
|  |  | 414.3 |  | Coronary atherosclerosis due to lipid rich plaque |
|  |  | 414.4 |  | Coronary atherosclerosis due to calcified coronary lesion |
|  |  | 414.8 |  | Other specified forms of chronic ischemic heart disease |
|  |  | 414.9 |  | Other forms of chronic ischemic heart disease |
|  | I25.9 | 414.8 |  | Other specified forms of chronic ischemic heart disease |
|  |  | 414.9 |  | Chronic ischemic heart disease, unspecified |
|  |  | 414.00 |  | Coronary atherosclerosis of unspecified type of vessel, native or graft |
|  |  | 414.01 |  | Coronary atherosclerosis of native coronary artery |
|  | I24.0 | 411.81 |  | Acute coronary occlusion without myocardial infarction |
|  | I24.8 | 411.89 |  | Other acute and subacute forms of ischemic heart disease |
|  | I24.9 | 411.89 |  | Other acute and subacute forms of ischemic heart disease |
| CAD in Death records |  |  |  | As for MI – where there is a CAD code in any position, the patient is classified as having CAD |

**ICD 9 codes were not used in UKB but are made available for studies where they are still used*
