# Supplementary material for: Reproducible disease phenotyping at scale: Example of coronary artery disease in UK Biobank
Source: PLoS One. 2022 Apr 5;17(4):e0264828. doi: 10.1371/journal.pone.0264828 (PMC8982857; doi:10.1371/journal.pone.0264828)
Supplement: S3 Table — (DOCX) [file pone.0264828.s003.docx]

**Supplementary Table 3: UKB available codes for CAD and MI diagnoses, medications, and procedures**

| **Data source** | **Field ID** | **Definition** |
| --- | --- | --- |
| ICD10 codes | 41270 | ICD10 diagnoses |
| CAD Operative Procedures | 41272 | Operative procedures (OPCS4) |
| Mortality | 40000  40001 | Date of death  Primary cause of death: ICD10 |
| Self-report CABG | 20004 | Self-report operative procedures (verbal interview) |
| Self-report PCI | 20004 | Self-report operative procedures (verbal interview) |
| Self-report angiogram | 20004 | Self-report operative procedures (verbal interview) |
| Self-report statin/aspirin use | 20003 | Self-report medications (verbal interview), see below for drugs/codes. |
| Statin/aspirin codes in UKB | 20003 | Statins:  Atorvastatin: (1141146234)  Crestor (10 mg tablet) (1141192414)  Eptastatin (1140910632)  Fluvastatin (1140888594)  Lescol (20mg capsule) (1140864592)  Lipitor (10 mg tablet) (1141146138)  Pravastatin (1140888648)  Rosuvastatin (1141192410)  Simvastatin (1140861958)  Velastatin (1140910654)  Zocor (10mg tablet) (1140881748)  Zocor heart-pro (10 mg tablet) (1141200040)  Aspirin:  Aspirin (1140861806, 1140864860, 1140868226)  Medication containing aspirin (1140868282, 1140872040, 1140882108, 1140882190, 1140882268, 1140882392, 1141163138, 1141164044, 1141167844) |
| SR MI | 6150;  20002 | Touchscreen questionnaire  Heart attack diagnosed by doctor (self-report)  Nurse led interview  Heart attack/ myocardial infarction (self-report) |
| SR CAD | 6150;  20002 | Touchscreen questionnaire  Angina diagnosed by doctor (self-report)  Nurse led interview  Angina (self-report) |
