# Supplementary material for: Reproducible disease phenotyping at scale: Example of coronary artery disease in UK Biobank
Source: PLoS One. 2022 Apr 5;17(4):e0264828. doi: 10.1371/journal.pone.0264828 (PMC8982857; doi:10.1371/journal.pone.0264828)
Supplement: S4 Table — (DOCX) [file pone.0264828.s004.docx]

**Supplementary Table 4: Baseline participant characteristics of coronary disease phenotype samples**

| **Baseline covariate** | **All CAD (N=37,476)** | **Incident MI**  **(N=4,621)** | **Prevalent MI**  **(N=4,900)** | **Self-Report MI**  **(N= 2,754)** | **Incident CAD without MI**  **(N=8,668)** | **Prevalent CAD without MI**  **(N=10,911)** | **Self-Report CAD without MI**  **(N=5,623)** | **CAD free Controls (N=465,155)** |
| --- | --- | --- | --- | --- | --- | --- | --- | --- |
| **Male** | 70.1% | 74.6% | 81.8% | 70.5% | 70.2% | 73.4% | 46.7% | 43.6% |
| **Age (years)** | 61.4 (6.3) | 60.4 (6.8) | 61.1 (6.3) | 61.5 (6.7) | 61.0 (6.3) | 62.3 (5.7) | 61.4 (6.4) | 56.1 (8.1) |
| **Type 2 Diabetes** | 16.2% | 12.4% | 17.0% | 17.3% | 14.6% | 19.1% | 14.8% | 4.4% |
| **SBP: Mean (SD)** | 142.3 (19.4) | 147.3 (19.3) | 136.7 (19.0) | 140.9 (18.8) | 146.2 (19.1) | 140.6 (19.2) | 141.0 (18.6) | 139.1 (19.0) |
| **Ever Smoker** | 72.4% | 81.3% | 79.5% | 76.2% | 70.4% | 68.9% | 65.9% | 53.0% |
| **Current smoker at baseline** | 26.4% | 36.3% | 31.0% | 31.1% | 25.4% | 21.1% | 22.4% | 15.6% |
| **BMI: Mean (SD)** | 28.8 (4.4) | 28.3 (4.2) | 28.8 (4.2) | 29.2 (4.6) | 28.6 (4.3) | 29.0 (4.3) | 29.2 (4.7) | 27.1 (4.3) |
| **Townsend deprivation index: (% in highest quartile of deprivation)** | 31.6% | 28.8% | 30.5% | 36.2% | 29.4% | 31.1% | 38.1% | 24.5% |
| **Statin use at baseline** | 61.5% | 29.7% | 89.3% | 61.5% | 37.0% | 82.7% | 60.1% | 12.7% |
