# Supplementary material for: Reproducible disease phenotyping at scale: Example of coronary artery disease in UK Biobank
Source: PLoS One. 2022 Apr 5;17(4):e0264828. doi: 10.1371/journal.pone.0264828 (PMC8982857; doi:10.1371/journal.pone.0264828)
Supplement: S1 File — (DOCX) [file pone.0264828.s005.docx]

**Online Supplement**

**Supplementary Methods**

***Algorithm Structure***

The algorithm first identified all individuals with a coded diagnosis of MI in EHR from secondary care or from mortality records (see supplementary methods for code lists) (**Figure 1)**. If no EHR coded diagnoses of MI was made, individuals were then classified as CAD without MI if they matched to code lists identifying presence of CAD, such as revascularization procedures (see supplement). Further classification based on date of enrolment categorized individuals as having prevalent (diagnoses prior to date of enrolment) or incident (occurring after enrolment) disease. If any MI or CAD without MI code was identified, the individual was censored from further classification.

Self-reported procedures relating to CAD, as opposed to self-reported diagnoses or tests, were included higher in the hierarchy on the assumption that patients would more reliably recall procedures than diagnoses. For two important procedures commonly used in cardiology, namely coronary angiography (a diagnostic test) and coronary angioplasty (a treatment) also known as percutaneous coronary intervention (PCI), we elected to improve specificity for presence of coronary disease by combining these procedures with prescription data for drugs routinely used if CAD had been identified. This was because an angiogram alone does not confirm CAD (as it can be normal), while the terms angiogram and angioplasty can frequently be confused by patients. We specified use of aspirin and statin from the UKB baseline questionnaire data for this step.

If individuals did not have one of the MI or CAD EHR codes, self-report diagnosis data for “heart attack” or “angina” was then used to classify remaining individuals as self-report MI and self-report CAD without MI, respectively. Finally, those who did not appear in any EHR source or self-report coronary disease were labelled as “never CAD” (if they since died) or “no CAD” (if they were still alive); and both groups were used as controls free from coronary disease. **Figure 1.**

All linked hospital admissions and mortality datasets were censored on November 1^st^, 2016, to maintain alignment and ensure completeness of follow-up across the available data sources.

The modular structure of the algorithm permits customization to allow researchers to generate specific phenotypes tailored to a particular question or analysis plan. For example, an analysis seeking to identify all patients with coronary revascularization, could be generated by starting directly at the relevant CAD level (“revascularisation in secondary care EHR”) passing the whole cohort through this filter, instead of at the top. This would permit inclusion of all people with revascularization procedures with or without a prior or incident MI, as these would no longer be selected out based on the hierarchical ordering of phenotypes. If a phenotype module is removed or re-ordered, the full cohort and algorithm needs to be re-run so that all participants are redistributed to a different phenotype.

On publication, algorithm-generated phenotypes will be returned to UK Biobank to be included in the data showcase for use by the research community and will in parallel be included in the HDR UK Phenotyping platform.
