# Supplementary material for: Reproducible disease phenotyping at scale: Example of coronary artery disease in UK Biobank
Source: PLoS One. 2022 Apr 5;17(4):e0264828. doi: 10.1371/journal.pone.0264828 (PMC8982857; doi:10.1371/journal.pone.0264828)
Supplement: Attachment — Submitted filename: CAD_PLOSONE_rebuttal.docx [file pone.0264828.s006.docx]

**Reviewer #1: This paper seeks to provide an algorithm for the classification of incident and prevalent CAD primarily using EHR coded data, supported by other coded data sources. There are several issues that need to be addressed to improve the utility of this work.**

We thank the reviewer for reviewing our paper and for the useful suggestions which we have addressed below.

**There was no comparison of the algorithm's classification to a "gold standard" classification. Hence, there was no "validation" in the understanding of most readers of this literature.**

We thank the reviewer for this comment which we have discussed internally. We agree that the use of the term “validation” has a very specific meaning for many readers and we have revised the manuscript in the Discussion section to further clarify this:

Algorithmic validation in the classical sense would include the expert, manual review of case notes and results from ECGs and the calculation of positive predictive value (PPV) and negative predictive value estimates (NPV). These data however are not available in large-scale population studies and EHR data such as the UK Biobank nor does the approach scale with large numbers of participants. We have therefore followed a robust evaluation approach which has been previously applied and validated in large scale EHR data that span care settings10 which provides multiple layers of evaluation through the replication of existing epidemiological findings and, in this case, a PRS for CAD. These multiple layers of complementary information provide further evidence towards the validity and robustness of the phenotyping algorithms presented here.

**It is not clear why the recommended approach of separating the dataset into the traditional testing and validation cohorts was not undertaken.**

We agree with the reviewer that had the purpose been to show an association with a marker or test a risk “model” (e.g. from machine learning) then it would be important to have a test and validation cohort.

However, as our goal was to create a rule-based, deterministic, phenotyping algorithm intended for use at scale, and then demonstrate its reliability, we felt it appropriate to classify all patients in one go. Given the number of sub-phenotypes that are generated by the algorithm, working with two smaller datasets would compromise the sample size in each phenotype category and thereby reduce power for any subsequent association studies. The loss of power for the prognostic and genetic associations (generating more imprecise estimates and wider CI) would then limit our ability to use the association analyses as a means of assuring the reader of the reliability of the phenotypes and algorithm itself.

**I do recognise that the classifications lead to outcome profiles that have some "fidelity" with the expected outcomes of incident and prevalent MI, and therefore provides some assurance, but this still remains a single sample problem, and does not meet there standards of using the term validation.**

We recognise the reviewers' concerns and have revised the manuscript as discussed above.

With regards to the single sample concern, we hope we have addressed this above, by distinguishing this algorithm as a rule-based classification tool that would work best with a large sample size, rather than a traditional outcome predictor which would indeed require a two sample approach.

**Please see and apply principles from the TRIPOD recommendations**

We thank the reviewer for this suggestion and would be happy to do this if the editors feel it is required, but our understanding is that the TRIPOD (Transparent reporting of a multivariable prediction model for individual prognosis or diagnosis) recommendations are for studies reporting on multivariable modeling, in order to improve reproducibility and standards for this type of work. For example a risk prediction model for CVD outcomes. We do not think our study falls into this category as there is no multivariable prediction modeling. Our work instead applies a sequential classification system to all participants in an algorithmic way by using carefully selected disease codes at each stage to filter out CAD cases and generate phenotypes that could be used by researchers in order to perform their studies.

**The use of coded data, rather than primary data sources (and the lack of validation) is problematic for the real clinical use of this algorithm. This should be recognised. This use of this algorithm is therefore useably for only cohort level or population level activities, rather than clinical (patient level) functions.**

Indeed the rationale for this work is primarily for use of this phenotyping algorithm in large scale research studies such as international biobanks where primary data sources are not easily available. Currently many researchers use a variety of ICD-9, ICD-10 or other terminology code combinations and self reported definitions of disease to define phenotypes of CAD for their epidemiological and association studies. Our work will enable a more methodical and referenced approach in order to help standardize research in these large cohorts and enable reproducible research.

We acknowledge that further work that is outside the context of this study is required to enable the clinical use of this algorithm in direct patient care.

**The inclusion of patient reported disease states is of uncertain value in this report. Some rationale for their inclusion should be provided.**

Self-reported diagnoses are frequently used by researchers, combined with EHR data sources, to define CAD and other phenotypes in UKB and other large scale studies. This leads to a larger number of cases, and subsequently increased statistical power for gene discovery. We have therefore also included these data in our study to evaluate the impact of their inclusion on analyses.

Our findings suggest that researchers should be careful when including self reported case definitions (e.g. self-report MI) as the actual ICD coded definitions of MI are likely to be more reliable; a similar pattern has been observed in other related studies (see Woodfield R. et al 10.1371/journal.pone.0137538) For gene discovery, including self-report CAD phenotypes may increase statistical power. In contrast, including self-report CAD data could bias mortality estimates.
